# Repeated aluminum ingestion alters human intestinal structure: focus on advanced 3D tissue models

**DOI:** 10.1007/s00424-026-03191-y

**Published:** 2026-07-09

**Authors:** Giulia De Negri Atanasio, Lorenzo Dondero, Francesca Tardanico, Giorgia Allaria, Erica Lertora, Giacomo Rosa, Chiara Barisione, Caterina Ivaldo, Francesca Rispo, Tommaso Filippini, Ilaria Demori, Sara Ferrando, Maria Cristina Gagliani, Katia Cortese, Mario Passalacqua, Jan Markus, Silvia Letasiova, Elena Grasselli

**Affiliations:** 1 Department of Earth, Environment and Life Science, University of Genoa, Genova, Italy; 2 Interuniversity Center for the Promotion of 3R Principles in Teaching and Research (Centro 3R), Pisa, Italy; 3 Angel Consulting srl, Via San Senatore 14, Milano, 20122 Italy; 4 National Biodiversity Future Center (NFBC), Piazza Marina, 61, Palermo, 90133 Italy; 5 Department of Surgical and Integrated Diagnostic Sciences, University of Genoa, Viale Benedetto XV,6, Genoa, 16132 Italy; 6 IRCCS Ospedale Policlinico San Martino, Largo Rosanna Benzi, 10, Genoa, 16132 Italy; 7 Environmental, Genetic and Nutritional Epidemiology Research Center (CREAGEN), Department of Biomedical, Metabolic and Neural Sciences, Section of Public Health, University of Modena and Reggio Emilia, Modena, Italy; 8 School of Public Health, University of California Berkeley, Berkeley, CA USA; 9 Department of Pharmacy, University of Genoa, Genova, Italy; 10 Department of Experimental Medicine, University of Genoa, Genoa, Italy; 11 MatTek Now Part of Sartorius, Bratislava, Slovakia; 12 National Center for the Development of New Technologies in Agriculture (Agritech), Napoli, Italy; 13 Istituto Nazionale Biostrutture e Biosistemi (INBB), Roma, 00136 Italy

**Keywords:** Aluminum, Repeated exposure, 3D in vitro models, Intestinal barrier, Microvilli length, Actin reorganization

## Abstract

Aluminum (Al³⁺) is a widespread environmental metal with potential effects on the intestinal epithelium. In this study, we evaluated the impact of dietary concentrations of aluminum on barrier integrity, histological organization, and gene expression in a 3D human intestinal tissue model. Tissues were exposed to increasing concentrations of aluminum (0, 5, 20, and 50 ppm) for 12 days to mimic environmental chronic exposure through ingestion. Transepithelial electrical resistance (TEER) measurements revealed a significant decrease only at the highest dose (50 ppm) on day 12, indicating a damage to the epithelial permeability. This was supported by a marked downregulation of OCLN and CLDN-5 expression, key genes involved in tight junction integrity. Aluminum permeation across the tissue was confirmed via Inductively Coupled Plasma—Atomic Emission Spectroscopy (ICP-AES), indicating dose-dependent penetration. Furthermore, gene expression analysis showed significant upregulation of MT-1E and MT-2 A (markers of cellular response to metal exposure) while MT-1 A remained unchanged. The present data demonstrate that repeated exposure to 50 ppm of Al3+ impacts intestinal barrier function and alters the tissue organization in the human intestinal epithelium.

**Graphical abstract:**

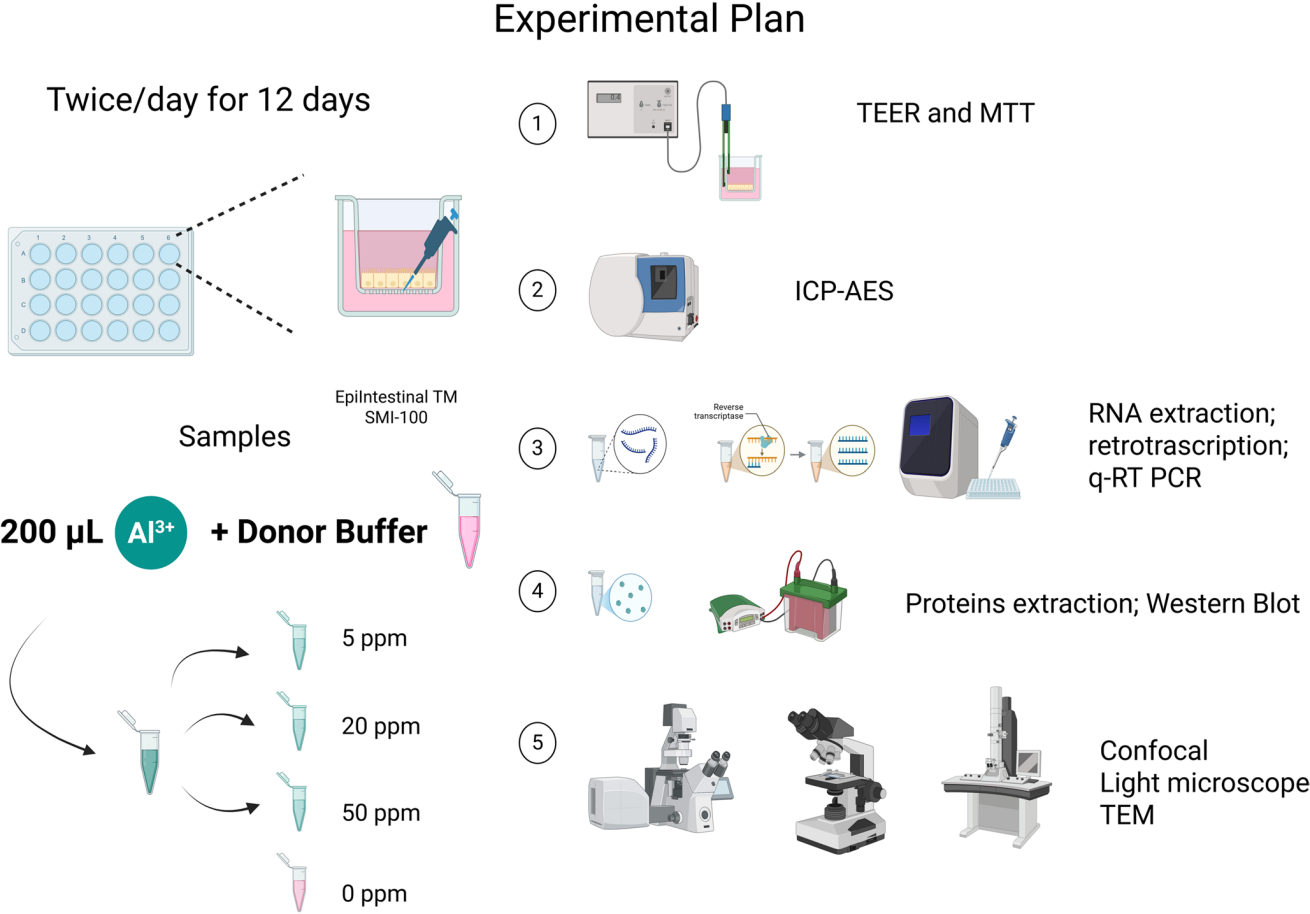

**Supplementary Information:**

The online version contains supplementary material available at 10.1007/s00424-026-03191-y.

## Introduction

Aluminum is the second most widely used metal globally and one of the most abundant elements in the Earth’s crust. Its unique physicochemical properties, including corrosion resistance, mechanical strength, and malleability, have led to widespread application across industrial, pharmaceutical, and food sectors. In the food industry, aluminum-based compounds are commonly employed as additives (e.g., anti-caking agents, leavening retardants, preservatives), while in pharmaceuticals they are used in vaccines as adjuvants and in antacids as active ingredients. Therefore, the primary route of exposure to aluminum for the general population is diet [1, 2]. Unprocessed foods typically contain less than 5 ppm of Al³⁺. Intermediate levels (5–10 ppm) are commonly found in products such as bread, cakes, and biscuits, where aluminum is used as an additive. Higher concentrations of Al³⁺ are observed in products such as coffee, cocoa, tea, and certain spices, with levels ranging from approximately 19 ppm up to 150 ppm (on a dry weight basis, in the case of some spices) [1, 3]. In addition, industrial food processing and packaging further increase aluminum levels in foods due to leaching from containers, the use of food additives, as well as from aluminum foils and utensils used for wrapping or cooking food [4].

The gastrointestinal tract functions as both the primary site of absorption and a major reservoir for aluminum accumulation. Experimental evidence suggests that a substantial fraction of ingested aluminum is retained within intestinal tissues, raising concerns about its potential impact on epithelial integrity and function. Although systemic absorption of aluminum following ingestion is relatively limited, cumulative intake has been associated with potential adverse effects on multiple organ systems. Compared to other biologically relevant metals such as sodium (Na⁺), calcium (Ca²⁺), and zinc (Zn²⁺), Al³⁺ carries a stronger positive charge and binds with high affinity to metal-binding amino acids such as arginine, histidine, and tyrosine. This binding induces protein oligomerization and conformational changes that hinder proteolytic degradation. Moreover, Al³⁺ interacts with phosphorylated amino acids, promoting aggregation of neurofilament proteins, microtubule-associated proteins, and other phosphorylated cytoskeletal proteins [5].

A body of literature has already described aluminum-induced alterations in intestinal cells and in vivo models. However, the effects of chronic exposure at dietary-relevant concentrations on intestinal morphology and physiology, particularly in advanced human 3D tissue systems, remain incompletely characterized [6–11]. Advanced human three-dimensional intestinal tissue models offer a physiologically relevant platform to investigate these effects, overcoming limitations of conventional two-dimensional cultures.

Given this context, the present study investigates the impact of repeated exposure to aluminum on the human gastrointestinal epithelium. Using advanced human three-dimensional intestinal tissue models, we evaluated morphological changes and protein expression after exposure to Al^3+^ concentrations representative of dietary intake, including high exposure dietary scenarios.

## Materials and methods

### Tissue experimental design

A human 3D in vitro small intestinal tissue model (EpiIntestinal SMI-100) was purchased from MatTek (Bratislava, Slovakia). First, tissues were incubated overnight to equilibrate the air-liquid conditions using an SMI-100-MM feeding medium (MatTek) at 37 °C, 5% CO_2_, and 95% RH. The experiments were performed for the next 12 days.

Every day, twice a day, tissues were placed in 24-well plates prefilled with 5 mL of basolateral/receiving buffer (Hank’s Balanced Salt Solution (HBSS, Gibco, Grand Island, NY, USA) supplemented with 0.2% D-glucose (Sigma Aldrich, Milan, Italy) and 10 mM HEPES (Gibco, Grand Island, NY, USA), pH 7.4).

200 µL of AlCl_3_ (Sigma Aldrich, Milan, Italy) diluted in apical/donor buffer (same composition as receiving buffer, but with pH 6.8) to final concentrations of 5 ppm, 20 ppm, and 50 ppm, were applied on tissue surface. HBSS solution free of AlCl_3_ was employed as control (0 ppm). Tissues were incubated for 2 h at 37° C and then placed back in the SMI-100-MM feeding medium.

### Trans-Epithelial Electrical Resistance (TEER) assay

To assess tissue integrity, the TEER assay was conducted before the treatment (time 0), and then every 2 days. Briefly, tissues were washed with sterile TEER buffer before being tested for TEER measurements with the Epithelial Voltohmmeter (EVOM; World Precision Instruments, Sarasota, FL, USA). Next, the resistance was adjusted to the surface area of the tissue and reported as Unit Area Resistance (Ω*cm^2^).

### Inductively Coupled Plasma—Atomic Emission Spectroscopy (ICP-AES) analyses

To analyze the total content of aluminum in the basolateral compartment of the tissue, the ICP-AES technique was carried out following the protocol described by Allaria et al. [12]. Briefly, samples were digested with nitric acid using microwave-assisted acid digestion at 180 °C for 50 min. Aluminum content was measured by ICP-AES on a Varian Vista PRO instrument at three aluminum-specific wavelengths (308.215, 394.401, and 396.152 nm) with lutetium (4 mg/L) as an internal standard. The procedural detection limit was 0.020 mg/L.

### Tissue viability

Following the exposure time, tissues were rinsed with Buffer B to remove any residual compounds. The samples were then transferred to a 24-well plate pre-filled with 0.3 mL of MTT solution (0.5 mg/mL) and incubated for 3 h. Post-incubation, the tissues were placed into 24-well plates containing 1 mL of isopropanol per well, with an additional 1 mL of isopropanol applied to the top of each insert to ensure complete extraction. The plates were incubated overnight at 4 °C. The following day, optical density was measured at 570 nm (Byonoy Absorbance 96, Germany), with all samples analyzed at least in triplicate.

### Histological analyses

EpiIntestinal inserts were washed in phosphate-buffered saline (PBS, 0.1 M, pH 7.4), and 4% buffered paraformaldehyde was gently added to both the wells and the inserts. After 1 h of fixation, the inserts were washed again in PBS, dehydrated through an ethanol series, cleared in BioClear (Bio-Optica, Milan, Italy), and embedded in paraffin. Tissues were then sectioned at 5 μm thickness using a Reichert-Jung 2035 rotary microtome (Leica Microsystems, formerly Reichert-Jung, Wetzlar, Germany) and mounted on glass slides. Sections were stained with hematoxylin and eosin (Bio-Optica, Milan, Italy) and examined under a Leica DMRB light microscope (Leica Microsystems, Wetzlar, Germany) equipped with a Moticam 3 + digital camera (Motic Europe, Barcelona, Spain). Cell vacuolation was semi-quantitatively evaluated by analyzing three histological sections per treatment. Vacuolated areas were manually counted and expressed as vacuole density, normalized to the epithelial area measured with ImageJ [13]. Briefly, the number of vacuolated cells per square millimeter of epithelium was calculated from the counts obtained in the linear sections.

### RNA extraction and quantitative real-time PCR

Total RNA was extracted using TRIreagent^®^ (Sigma-Aldrich Milan, Italy) according to the manufacturer’s instructions as previously described [14]. The iScript cDNA Synthesis Kit (Bio-Rad) was used to convert the extracted RNA to cDNA according to the manufacturer’s instructions. Quantitative RT-PCR was performed in triplicate using IQ supermix (Bio-Rad) on CFX96 Real-Time system (C1000 Thermal Cycler; Bio-Rad) according to the following parameters: 95° C x 3min, 40 cycles at 95° C x 15s and 60° C x 40s. Expressions of the following biomarkers were evaluated: different isoforms of metallothioneins (MT1A, MT1E, and MT2A), occludin (OCLN) and claudin-5 (CLDN-5). Glyceraldehyde-3-phosphate dehydrogenase (GAPDH) expression was used as housekeeping gene (GAPDH Fwd: 5’- GACCCCTTCATTGACCTCAAC-3’; GAPDH Rev: 5’- CGCTCCTGGAAGATGGTGATGGG − 3’) using SYBR Green Supermix (Bio-Rad) on CFX96 Real-Time system (C1000 Thermal Cylcer; Bio-Rad).

### Protein extraction and western blot analysis

Proteins were extracted by lysing the cells with 10X RIPA buffer [20 mM Tris-HCl (pH 7.5), 150 mM NaCl, 1 mM Na₂EDTA, 1 mM EGTA, 1% NP-40, 1% sodium deoxycholate, 2.5 mM sodium pyrophosphate, 1 mM β-glycerophosphate, 1 mM Na₃VO₄, and 1 µg/mL leupeptin (Cell Signalling Technology)]. 400 µL of 1X RIPA buffer supplemented with 10% sodium deoxycholate was added and samples were incubated for 10 min. Samples were then homogenized and centrifuged at 12,000 × g for 20 min at 4 °C. The resulting supernatant was collected in a new tube for subsequent analysis. Protein concentration was determined using the BCA assay (Pierce BCA Protein Assay Kit, Thermo Fisher Scientific, Rockford, USA). 20 µg of total protein lysates were resolved by electrophoresis on precast polyacrylamide gels (NuPAGE 8–16% Bis-Tris, 1.0–1.5 mm, Mini Protein Gels, Invitrogen) and transferred onto membranes following standard Western blot protocols. Immunodetection was performed using a goat anti- total actin polyclonal antibody (Santa Cruz Biotechnology) and a rabbit polyclonal anti-Histone H3 antibody (Cell Signalling Technology) as a reference for protein normalization. Bands of expression were detected using an ECL substrate (Bio-Rad) and visualized with the UVITEC Q9 Alliance imaging system (Cambridge).

### Confocal microscopy

Following treatment, EpiIntestinal inserts were fixed with 4% paraformaldehyde for 30 min at room temperature and subsequently permeabilized with 0.1% Triton X-100. Nuclei were stained with 1 µg/mL 4′,6-diamidino-2-phenylindole (DAPI, Thermo Fisher Scientific, Monza, MB, Italy), and F-actin was visualized using Phalloidin-FITC. Imaging was performed using a Nikon AX R confocal microscope equipped with a PLAN APO 25×/NA 1,05 silicon oil immersion objective (Nikon Europe B.V., Stroombaan 14, 1181 VX Amstelveen, The Netherlands). Excitation and emission settings were as follows: DAPI 405 nm excitation/420–500 nm emission, Phalloidin-FITC 488 nm excitation/500–550 nm emission. The pinhole size was set to 1 Airy Unit at 488 nm. Images were acquired at a resolution of 2048 × 2048 pixels with a pixel size of 0.2 μm. Sequential scanning was employed to minimize spectral overlaps and avoid fluorescence channel crosstalk.

### Transmission Electron Microscopy (TEM)

A total of 10 samples were analyzed after exposure to aluminum at concentrations of 5, 20, or 50 ppm, or left untreated. Tissues were gently rinsed in PBS and fixed in 2.5% glutaraldehyde in 0.1 M sodium cacodylate buffer (pH 7.4) for 1 h at room temperature. Following two washes in the same buffer, samples were post-fixed with 1% osmium tetroxide for 1 h. Dehydration was performed through a graded ethanol series (70%, 95%, 100%), followed by propylene oxide. Samples were then infiltrated with increasing concentrations of Epon resin in propylene oxide and embedded flat in resin-filled capsules. Polymerization was carried out at 60 °C for 48 h. Polymerized blocks were trimmed, and ultrathin Sects.  (60–70 nm) were obtained using a Leica Ultracut ultramicrotome and collected on copper grids. Sections were contrasted with uranyl acetate and lead citrate according to standard procedures. Transmission electron microscopy was performed using a Hitachi HT7800 TEM operating at 100 kV and equipped with a Megaview III digital camera. Images were acquired and analyzed using Radius 2.0 software. Morphometric analysis of microvilli length was conducted on 10 randomly selected micrographs per condition (20,000× magnification, 2 × 2 image alignment) using the line measurement tool in Radius 2.0. Microvilli length was expressed in nanometers and presented as histograms displaying individual measurements.

### Statistical analysis

All experiments were performed in triplicate (*n* = 3) within each experiment. To ensure biological reproducibility, the entire experimental plan was replicated across three different production batches of EpiIntestinal tissues. Statistical analyses were performed using the GraphPad Prism software (v10.0). One-way ANOVA followed by Tukey’s multiple comparison test was used for multiple groups. A significance level of *P* < 0.05 was considered indicative of statistically significant differences.

## Results and discussion

### TEER measurements and OCLN and CLDN-5 gene expression in EpiIntestinal tissues treated with aluminum

**Fig. 1:**
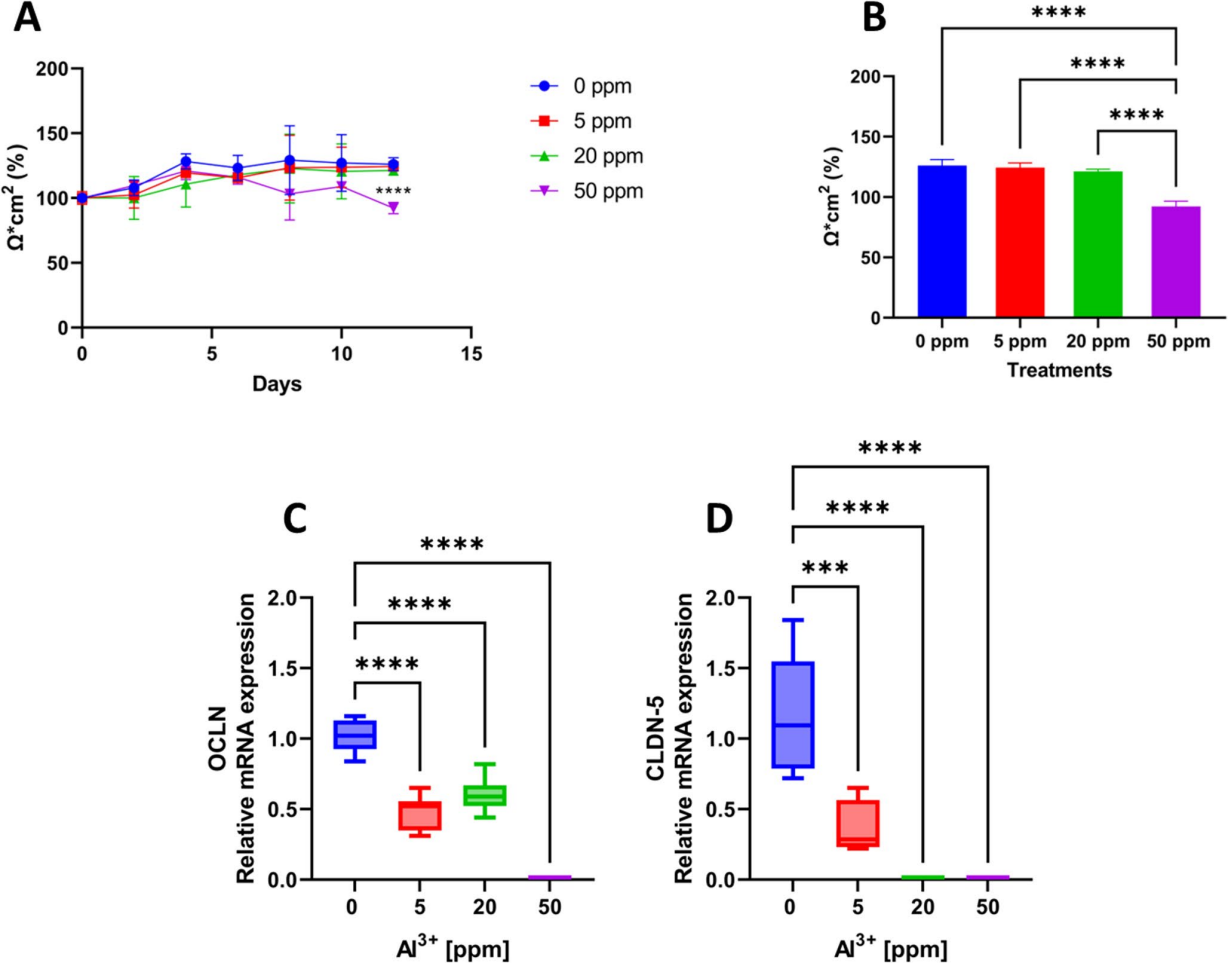
TEER Measurements and OCLN and CLDN-5 Gene Expression in the EpiIntestinal Tissue Models Exposed to Aluminum. (**A**) TEER measurements of 3D EpiIntestinal tissues treated with 0, 5, 20, and 50 ppm of Al³⁺ over 12 days. (**B**) TEER measurements on Day 12, comparing tissues treated with 0, 5, 20, and 50 ppm of Al³⁺. Statistical significance is indicated by symbols (****p < 0.0001 vs. 50 ppm). Data are presented as mean ± SD (*n* = 3) (**C**) and (**D**) relative gene expression of Occludin and Claudin-5 after 12 days Al³⁺ exposure. In the box-and-whisker plots the horizontal line indicates the median, the box reflects values from the first to the third quartile, and the whiskers represent the furthest points that are not outliers. The significant differences are indicated by symbols on bars (**** *p* ≤ 0.0001, *** *p* ≤ 0.001, vs. 0 ppm) (*n* = 3)

To determine the effect of Al³⁺ on barrier function, TEER was measured in EpiIntestinal tissue following treatment. As shown in Fig. 1(A), no statistically significant differences in TEER values were observed at any concentration or time point when compared to the 0 ppm except for the final day of treatment where a statistically significant difference was observed in tissues exposed to the highest Al³⁺ concentration, as shown in Fig. 1(B), suggesting an impact on epithelial permeability.

The results were also supported by the gene expression analysis of *OCLN* and *CLDN-5*, as a proxy of tight junction (TJ) integrity [15, 16]. After 12 days of treatment, tissues exposed to Al³⁺ showed a significant reduction in the mRNA expression levels of both genes (Fig. 1C and D).

### Inductively Coupled Plasma—Atomic Emission Spectroscopy (ICP-AES) analyses and MT1A, MT1E and MT2A gene expression in Epiintestinal tissues treated with aluminum

**Fig. 2:**
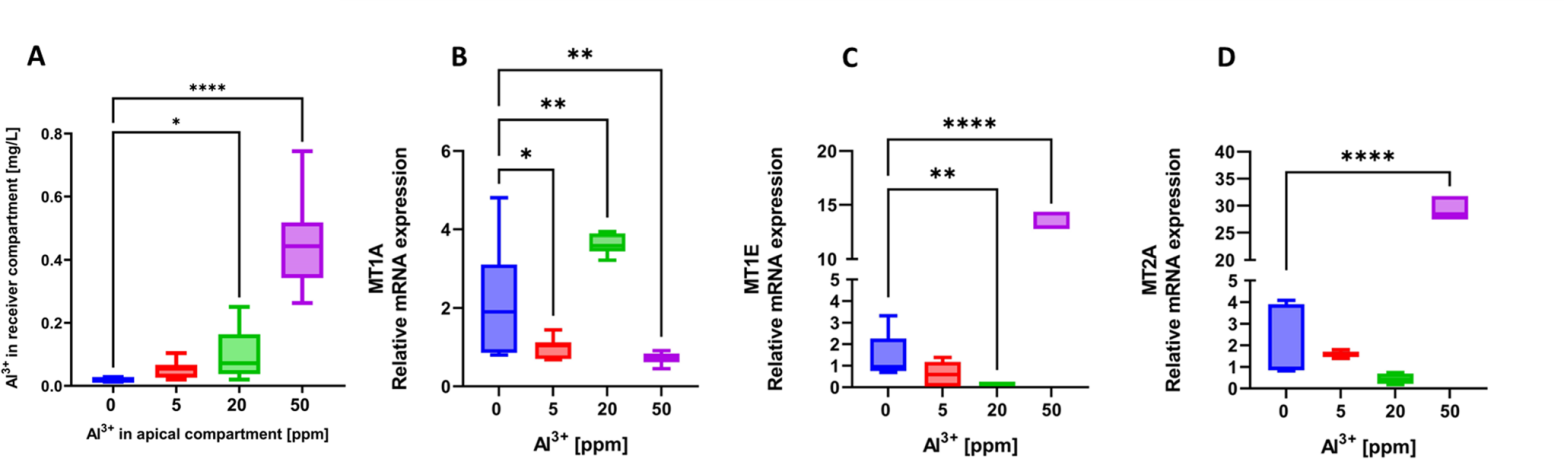
ICP-AES analysis and MT1A, MT1E, MT2A gene expression in the EpiIntestinal Tissue Models Exposed to Aluminum. (**A**) Assessment of Al^3+^ trespass to the intestinal mucosa. (**B**), (**C**) and (**D**) relative gene expression of MT-1 A, MT-1E, and MT-2A after 12 days Al³⁺ exposure. In the box-and-whisker plots the horizontal line indicates the median, the box reflects values from the first to the third quartile, and the whiskers represent the furthest points that are not outliers. The significant differences are indicated by symbols on bars (*** *p* ≤ 0.0001, ** *p* ≤ 0.01, * *p* ≤ 0.05 vs. 0 ppm) (*n* = 3)

Inductively Coupled Plasma (ICP) analysis was employed to quantify the amount of aluminum in basolateral medium, to evaluate its ability to cross the epithelial barrier. The results demonstrated a correlation between the applied aluminum concentration and the amount of metal detected by plasma spectrometry, suggesting a dose dependent passage of aluminum across the tissue barrier (Fig. 2A).

To investigate the cellular response to aluminum exposure, gene expression analysis was carried out using quantitative PCR (qPCR) targeting the MT1A, MT1E, and MT2A isoforms of metallothioneins. Tissues treated with 50 ppm of aluminum showed a statistically significant increase in the expression of MT2A and MT1E, while MT1E was significantly downregulated at 20 ppm (Fig. 2C, D), compared to untreated controls. On the other hand, the MT1A isoform was significantly upregulated at 20 ppm, while it was significantly downregulated at both 5 and 50 ppm (Fig. 2B). Metallothioneins are proteins involved in metal ion homeostasis, and their upregulation is often associated with cellular protection mechanisms against oxidative stress.

### Histological analyses of EpiIntestinal tissues treated with aluminum

**Fig. 3:**
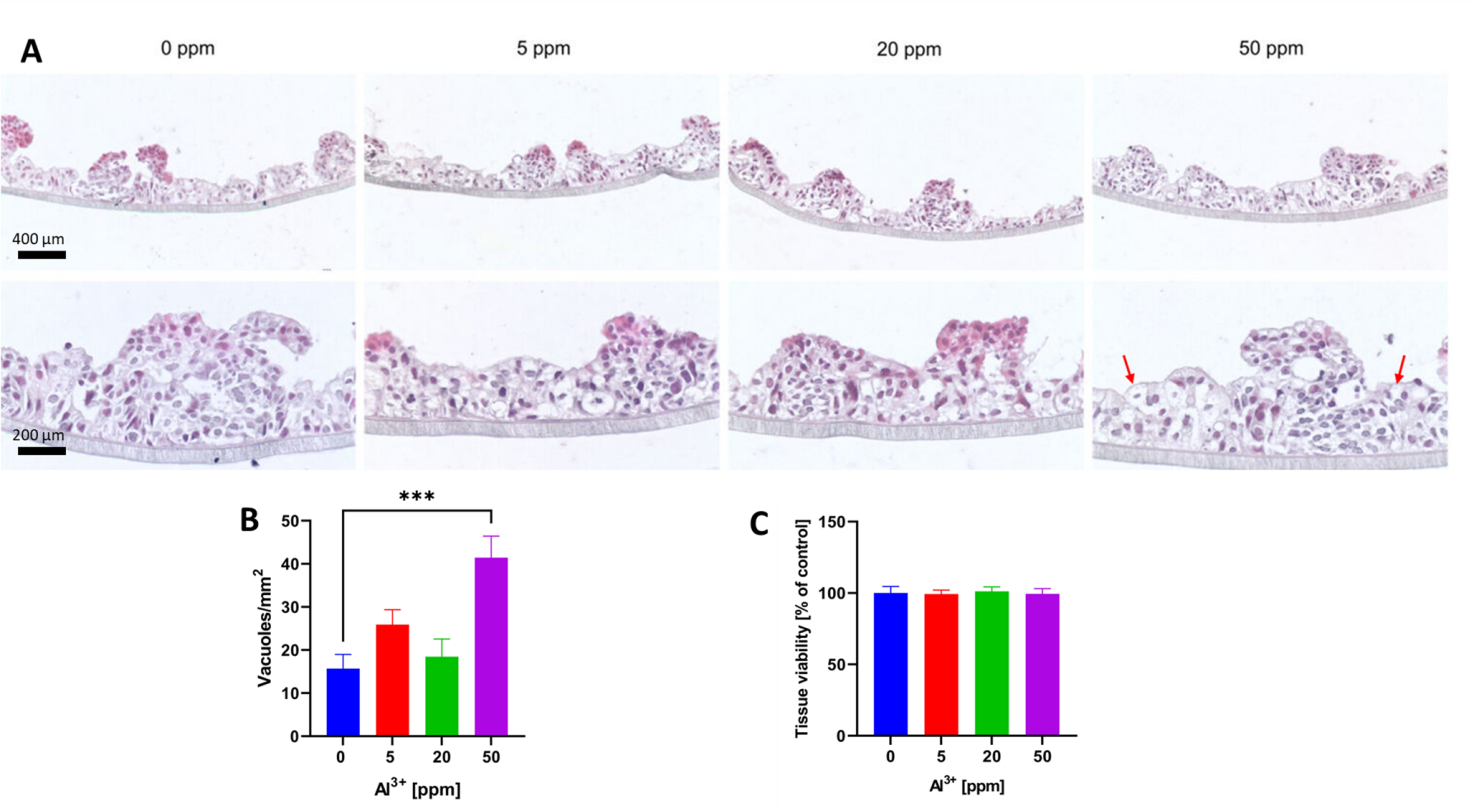
Evaluation of vacuolization in the EpiIntestinal Tissue Models Exposed to Aluminum. (**A**) Sections of intestine at two magnifications, exposed to different concentrations of aluminum and stained with hematoxylin and eosin (magnification 100X and 200X) after 12 days Al³⁺ exposure. Arrows indicate some vacuolated cells. (**B**) Density of vacuoles per mm^2^ for the concentrations tested following 12 days exposure. (**C**) Tissue viability after 12 days of treatment with different concentrations of Al^3+^. Statistical significance was evaluated using one-way ANOVA (***p < 0.001 vs. 50 ppm)

Light microscopy analysis of the histological sections indicated that none of the tested aluminum concentrations caused major alterations in the overall morphology of the inserts. The tissue architecture appeared largely preserved, with no evident signs of disruption or degeneration in most samples. However, at 50 ppm aluminum, distinct histological changes were observed. Vacuolated enterocytes were visible and frequently distributed throughout the tissue. These morphological alterations appeared to affect the organization of the tissue, reducing its compactness and potentially indicating early signs of cellular stress or damage (Fig. 3.A). ANOVA on the average vacuole density estimates yielded significant differences among concentrations: F (2, 15) = 17.2, *p* < 0.001. A *post hoc* Tukey test showed that vacuole density of inserts exposed to 50 ppm aluminum was significantly higher compared to other treatments (Fig. 3B). Tissue viability was also investigated, the treatment with Al^3+^ did not result in any significant impact on the tissue viability at any of the concentrations tested (5, 20, and 50 ppm). Statistical analysis confirmed that viability remained comparable to the control group, indicating no cytotoxic effect under the experimental conditions.

### Confocal images and actin detection of EpiIntestinal tissues treated with aluminum

To investigate the effects of Al³⁺ exposure on the actin cytoskeleton, we performed confocal fluorescence top-down imaging of EpiIntestinal tissues stained with phalloidin (to visualize F-actin) and DAPI (to label the nuclei). In control tissues (0 ppm aluminum), phalloidin staining revealed well-organized actin filaments and a homogenous, intact epithelial structure. DAPI staining showed uniformly shaped nuclei, consistent with preserved cellular morphology.

Similar F-actin organization was observed in tissues treated with 5 ppm and 20 ppm aluminum, indicating that low to moderate concentrations of Al³⁺ did not have an impact on the actin cytoskeleton. Under these conditions, actin filaments remained well-structured.

**Fig. 4:**
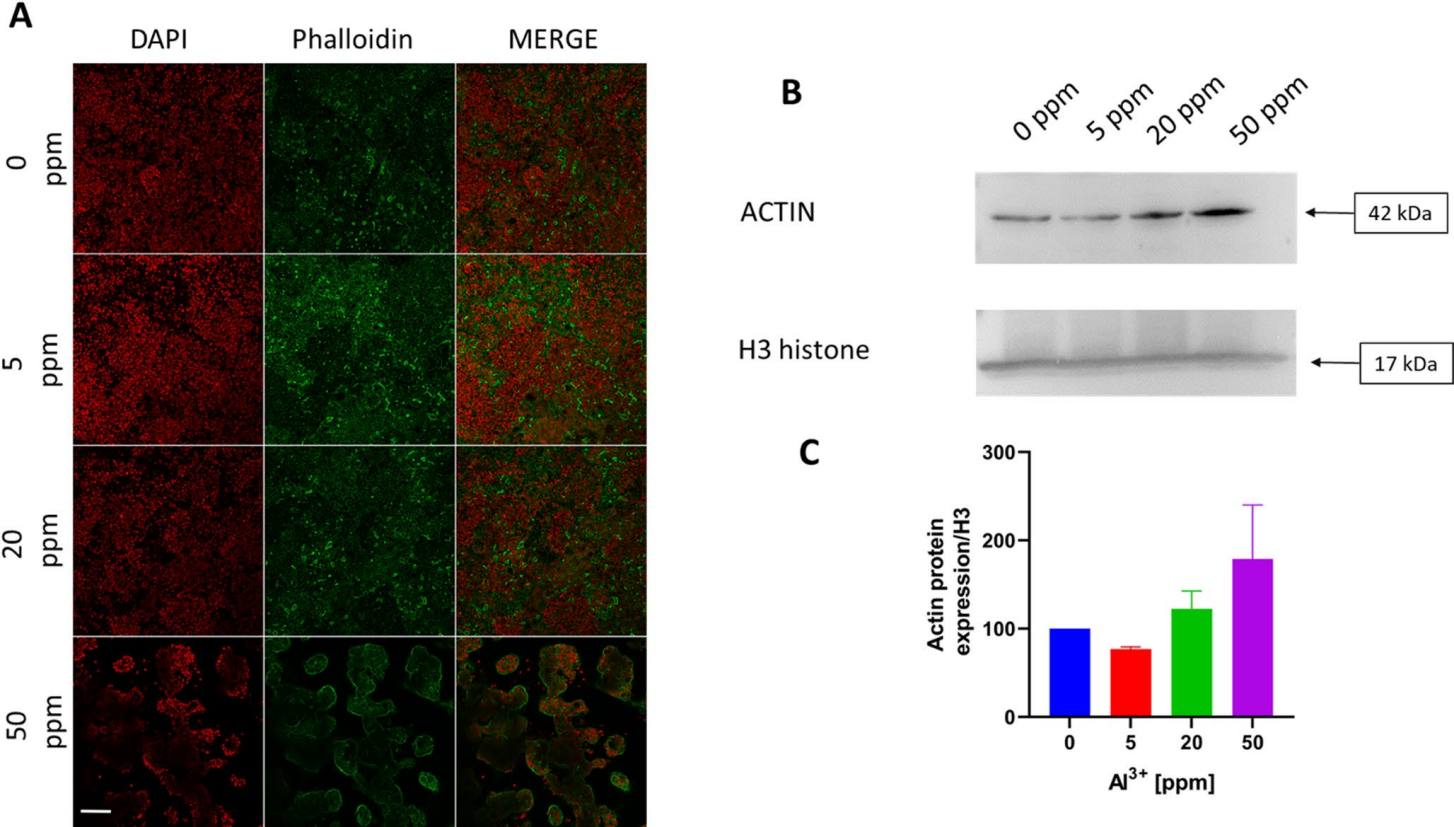
Dose-Dependent Effects of Aluminum on F-Actin Distribution and Expression in the EpiIntestinal Tissue Models Exposed to Aluminum. (**A**) Representative Confocal images of 3D EpiIntestinal tissues treated with 0, 5, 20, and 50 ppm of Al³⁺ over 12 days. Red indicates nuclei stained with DAPI and green indicates F-actin stained with phalloidin-FITC (scale bar 100 μm). (**B**), (**C**) Representative Western blot analysis of 3D EpiIntestinal tissues treated with 0, 5, 20, and 50 ppm of Al³⁺ over 12 days

However, exposure to 50 ppm aluminum induced marked changes in actin distribution. Phalloidin staining confirmed compromised tissue integrity and revealed a noticeable F-actin accumulation at the apical surface of EpiIntestinal tissue. These structural changes are illustrated in representative confocal images shown in Fig. 4A.

Western blot analysis suggested a trend toward increase in total actin expression, with the highest levels observed at 50 ppm aluminum; however, these differences were not statistically significant (Fig. 4B, C).

Overall, these results suggest a concentration-dependent effect of Al³⁺ on cytoskeletal organization, with significant actin remodeling occurring at 50 ppm, which may contribute to the epithelial integrity or function.

### Transmission Electron Microscopy (TEM) of EpiIntestinal tissues treated with aluminum

**Fig. 5:**
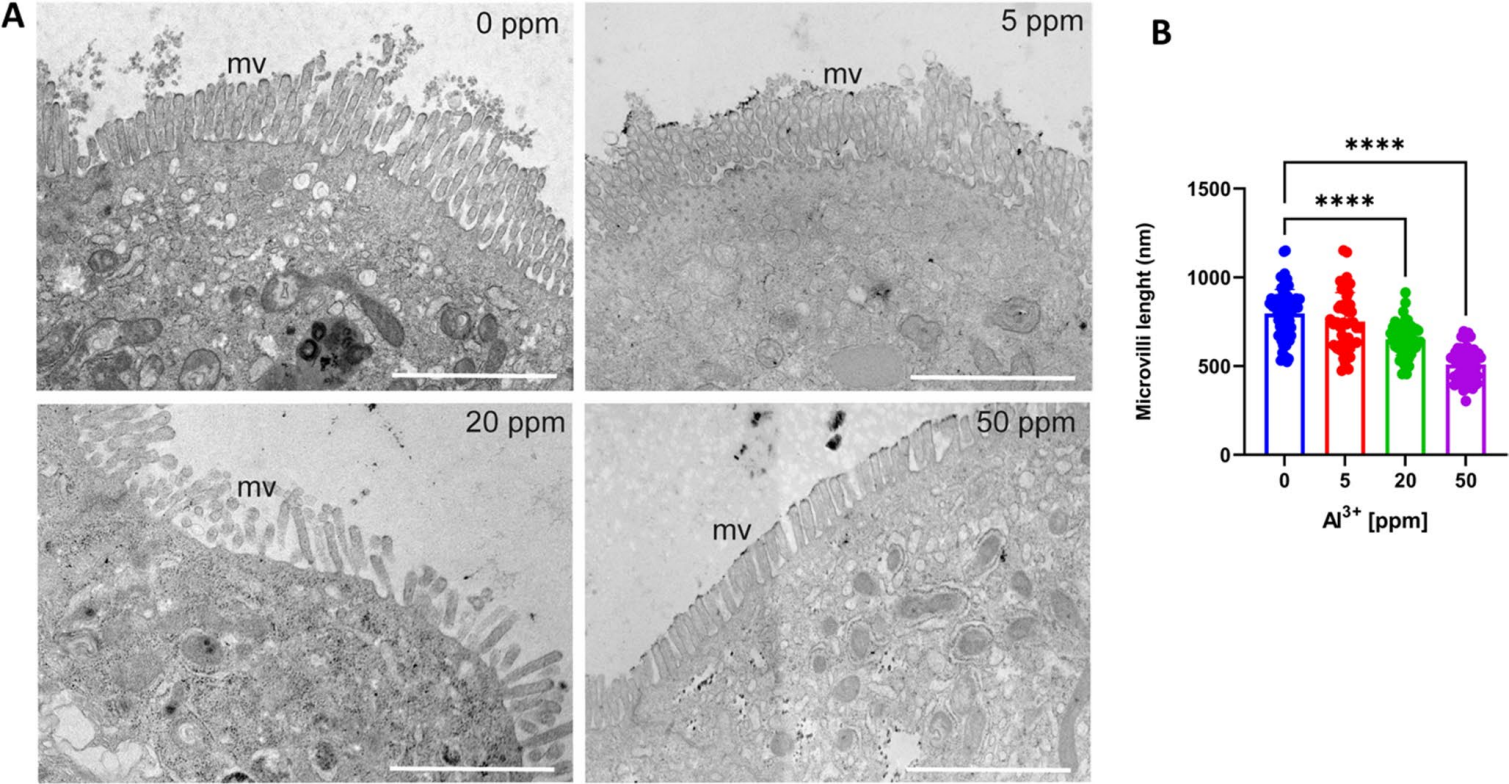
Ultrastructural Analysis of Microvilli Length in the EpiIntestinal Tissue Models Exposed to Aluminum after 12 days of treatment. (**A**) Representative transmission electron microscopy (TEM) images of EpiIntestinal tissues either untreated (0 ppm) or exposed to aluminum at 5, 20, and 50 ppm. A progressive reduction in microvilli (mv) length is observed with increasing aluminum concentrations. Scale bars: 2 μm. (**B**) Quantification of microvilli length measured from 10 random TEM fields per condition (20,000× magnification, 2 × 2 image alignment). Each dot represents an individual measurement; data are expressed in nanometers. The significant differences are indicated by symbols on bars (****p < 0.00001; ***p < 0.0001)

To evaluate the impact of aluminum exposure on intestinal epithelial morphology, we performed transmission electron microscopy (TEM) on EpiIntestinal tissues exposed to increasing concentrations of aluminum (5, 20, and 50 ppm) compared to untreated controls. TEM images revealed a progressive reduction in the length of apical microvilli with increasing aluminum concentration (Fig. 5A). Morphometric analysis confirmed a significant decrease in microvilli length, quantified on 10 randomly selected images per condition. As shown in the histogram (Fig. 5B), microvilli shortening was dose-dependent, with statistically significant differences observed at 20 and 50 ppm compared to control (****p < 0.00001; ***p < 0.0001).

## Conclusion

In this work, we provide evidence that aluminum concentrations relevant to high dietary exposure scenarios of aluminum can impair the intestinal barrier in a 3D human reconstructed intestinal model, which better mimics human intestinal physiology than conventional monolayer cultures [17]. The use of 3D intestinal tissue models represents a significant advancement in research, as they closely replicate the structural and functional complexity of the human intestinal epithelium. Produced under Good Manufacturing Practice (GMP) standards, these models provide a robust and reproducible platform for toxicological studies. Importantly, their use aligns with the principles of New Approach Methodologies (NAMs), which promote innovative, human-relevant alternatives to traditional animal-based models. NAMs not only enhance the ethical profile of research but also improve its scientific quality and translational potential by focusing on systems that better reflect human physiology. The most pronounced effects were consistently observed at the highest concentration tested (50 ppm). While lower concentrations are representative of common dietary exposure, the 50 ppm condition is more appropriately considered as relevant to highly exposed consumers and high-end exposure scenarios.

Aluminum was administered to the EpiIntestinal models at different doses (5–50 ppm), consistent with levels that can be ingested through the diet, following a food intake-like model of twice-daily administration (2-hour treatments) for 12 days. Under these conditions, we observed a reduction in intestinal permeability at the highest dose tested (50 ppm), accompanied by decreased expression of the TJ proteins OCLN and CLDN-5. These markers were selected as representative indicators of tight junction alterations in combination with functional TEER measurements. Although tight junction regulation involves multiple claudins and associated proteins, the concordance between gene expression changes and TEER reduction supports a consistent interpretation of barrier impairment. The altered barrier integrity at 50 ppm was associated with an upregulation of metallothionein isoforms MT1E and MT2A. Vacuolization was observed within the epithelial layer, particularly at 50 ppm. Given that the EpiIntestinal model is primarily composed of differentiated enterocyte-like cells, these vacuolated elements are attributable to the epithelial compartment. In this context, vacuolization is interpreted as a morphological indicator of cellular stress/injury. Moreover, the absence of Alcian Blue/PAS-positive cells in the control EpiIntestinal epithelium (data not shown) indicates that mucus-producing cells are not present in this model, consistent with the manufacturer’s characterization. Therefore, the vacuolated cells observed in the treated tissues are unlikely to represent goblet cells and are more reasonably attributable to enterocyte-like cells. Additionally, we found that aluminum exposure leads to a disorganization of actin filaments within intestinal cells, resulting in a more clustered pattern. This disorganization is associated with shorter microvilli and an overall increase in total actin levels, as confirmed by Western blotting. These effects were not accompanied by changes in cell viability, as assessed by the MTT assay, indicating that the observed alterations were not associated with overt cytotoxicity.

Our findings suggest that aluminum alters the intestinal barrier by downregulating TJ proteins and altering actin filament organizations. TJs—formed by proteins such as occludins, claudins, and junctional adhesion molecules (JAMs)—seal neighboring epithelial cells and regulate paracellular permeability. These junctions are anchored to the actin cytoskeleton through adaptor proteins like zonula occludens-1 (ZO-1), ZO-2, and ZO-3. The integrity and dynamic regulation of TJs thus depend critically on the cytoskeleton. The observed shortening of microvilli can also be attributed to actin disorganization. Each microvillus is supported by a tightly packed bundle of actin filaments, stabilized by actin-associated proteins such as villin and fimbrin, which maintain microvillar structure and function. This complex actin network not only shapes microvilli but also plays a central role in sustaining the epithelial barrier [18–20]. Although metal exposure is commonly associated with the disruption or loss of F-actin structure, certain metals can paradoxically increase F-actin expression and polymerisation in response to stress and as a compensatory mechanism, particularly at low or sub-toxic doses [21]. This is in line with the trend of increase, even if not statistically significant, in total actin detected in our study, that likely reflects a compensatory attempt by the tissue to maintain structural and functional integrity despite aluminum-induced alterations to cytoskeletal organization. (Alternatively, this increase may also reflect changes in the dynamics of actin turnover).

Since aluminum belongs to the class of metals, some of its effects may be interpreted in the broader context of metal-induced intestinal alterations, as several metals are known to impair epithelial barrier function and cytoskeletal organization. Metals act as pro-oxidants that can compromise barrier integrity and trigger inflammation. Previous studies have shown that exposure to heavy metals such as arsenic, cadmium, copper, manganese, mercury and nickel reduces epithelial barrier function by disrupting TJ, partly through early alterations in β-actin synthesis and organization—often at concentrations that do not yet affect cell viability [22]. Similar effects have been observed with other pro-oxidant stimuli that cause actin cytoskeleton remodeling. The mechanism behind aluminum’s effect may thus involve its pro-oxidant properties, which lead to protein adduct formation and actin rearrangement, as reported for other oxidative stress conditions [23]. Previous in vitro studies using intestinal monolayers have demonstrated that (1) an intact cytoskeleton—particularly actin and tubulin—is essential for maintaining barrier integrity [24–30]; (2) oxidants can disrupt intestinal barrier function [31–33]; (3) this loss of function requires oxidative damage to actin and tubulin; (4) full or partial disruption of these structures leads to barrier breakdown; and (5) protecting the cytoskeleton can mitigate oxidant-induced damage. Taken together, these findings support the idea that oxidative stress, when uncompensated, leads to defective barrier function and shortening of microvilli, ultimately reducing the absorptive surface area. In line with this, no significant changes in IL-6 and IL-8 secretion were observed under the present experimental conditions (data not shown), suggesting that aluminum exposure primarily affects epithelial barrier integrity and cytoskeletal organization rather than inducing a pronounced pro-inflammatory response.

Our results demonstrated that aluminum was able to modulate MT isoform expression. MTs may help limit aluminum’s potential toxicity in all tissues including the intestine. Although aluminum is not a primary target for MTs, these small, cysteine-rich proteins can indirectly influence its behavior by binding other metals, modulating their absorption and storage, and regulating inflammatory responses.

Damage to the intestinal barrier has been observed after aluminum ingestion, suggesting potential compromise of gut integrity and systemic exposure [34]. Understanding these physiological interactions is essential for evaluating the health impact of dietary aluminum exposure.

In conclusion, our results demonstrate that aluminum, at concentrations commonly found in food, can significantly affect the structure and function of the small intestine, highlighting potential health risks associated with chronic dietary exposure. Our data support a mechanistic model in which aluminum-induced stress reorganizes F-actin, leading to microvilli shortening and tight junction disassembly.

## Supplementary Information

Supplementary Material 1.

Supplementary Material 2.

Supplementary Material 3.

Supplementary Material 4.

Supplementary Material 5.

Supplementary Material 6.

Supplementary Material 7.

## Data Availability

The data that support the findings of this study are available from the corresponding author upon reasonable request.
