# Supplementary figures and images for: Repeated aluminum ingestion alters human intestinal structure: focus on advanced 3D tissue models
Source: Pflugers Arch. 2026 Jul 9;478(7):61. doi: 10.1007/s00424-026-03191-y (PMC13346327; doi:10.1007/s00424-026-03191-y)

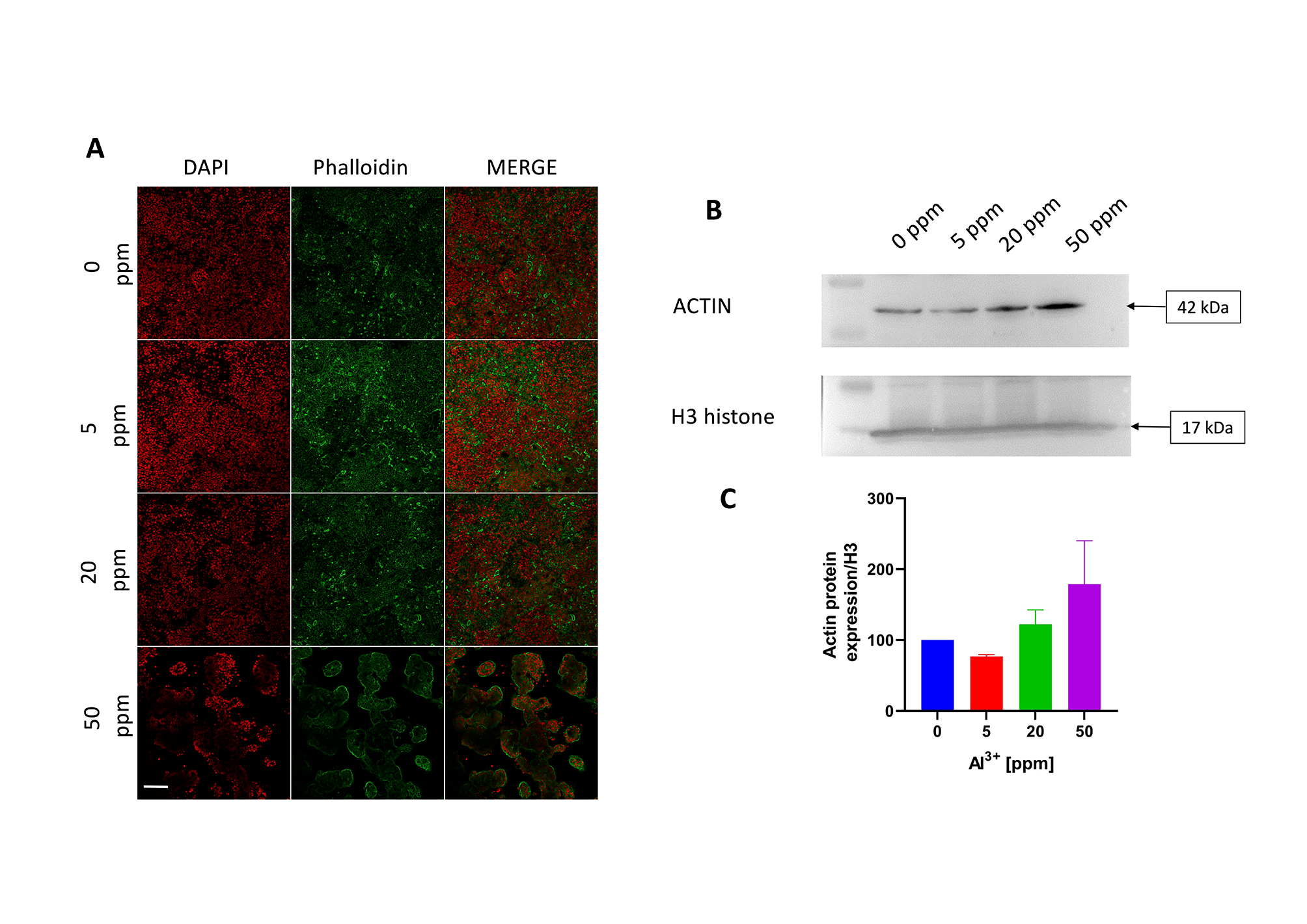

Supplement: Supplementary file 1 — Supplementary Material 1. [file 424_2026_3191_MOESM1_ESM.png]

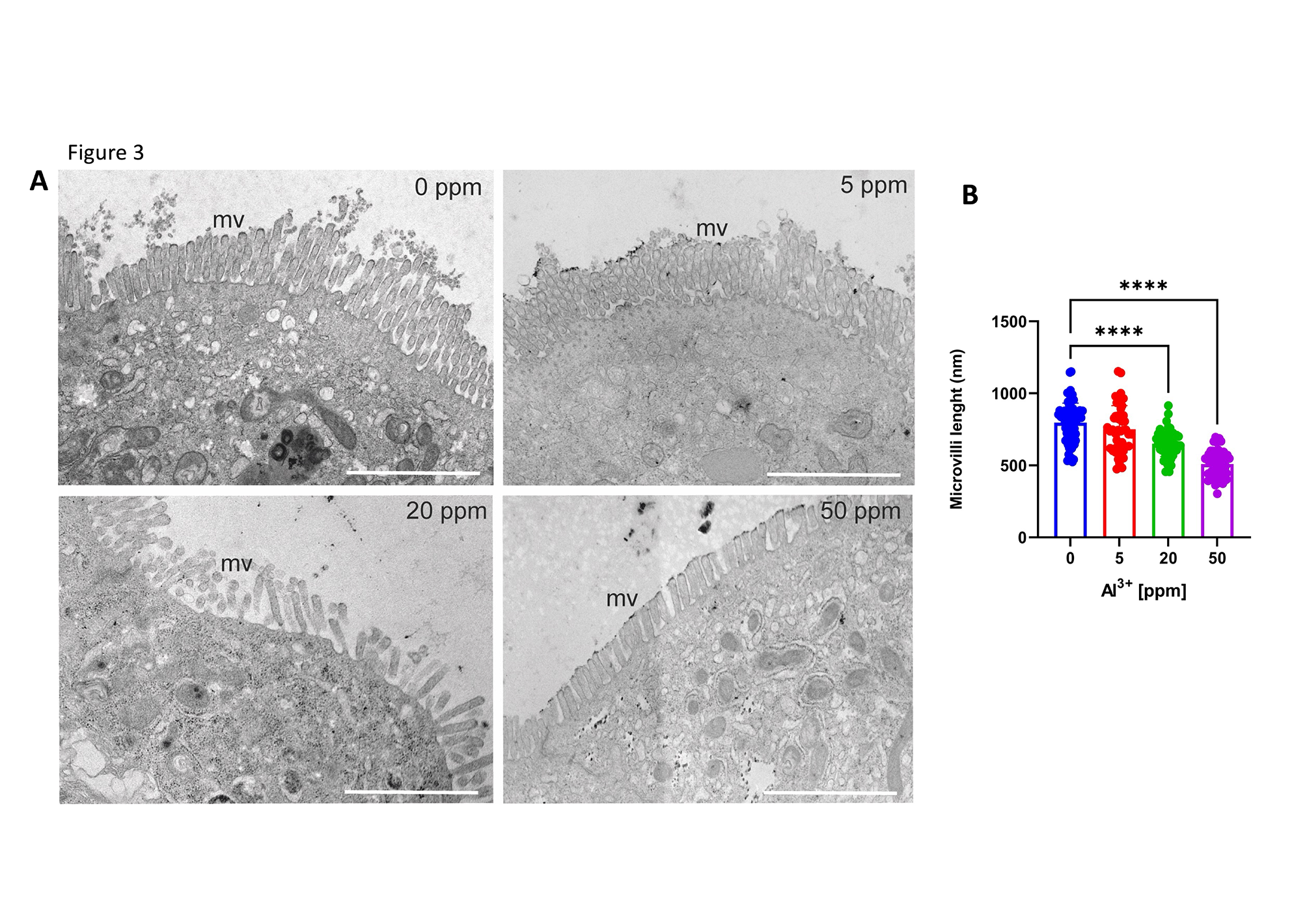

Supplement: Supplementary file 2 — Supplementary Material 2. [file 424_2026_3191_MOESM2_ESM.png]

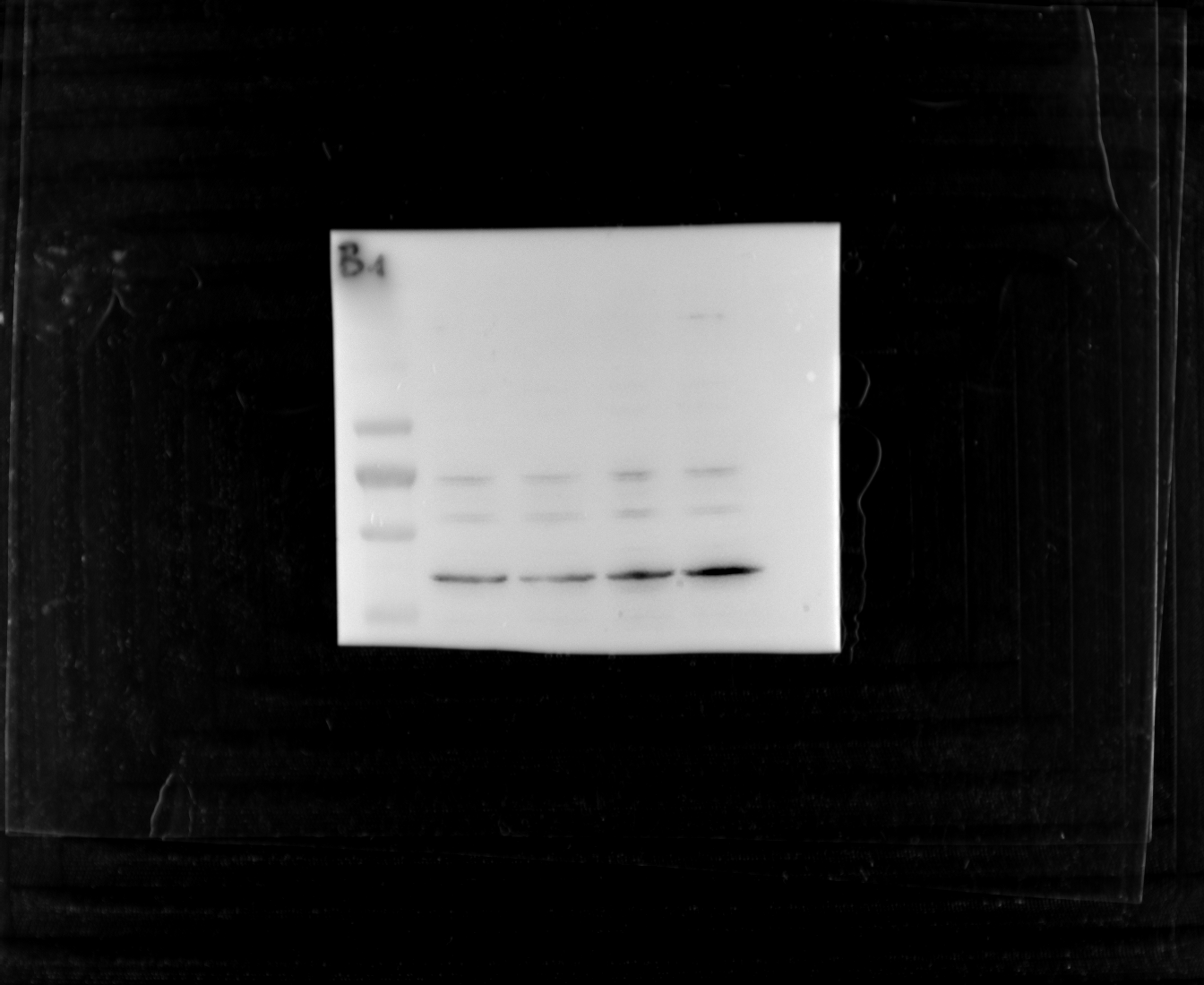

Supplement: Supplementary file 3 — Supplementary Material 3. [file 424_2026_3191_MOESM3_ESM.png]

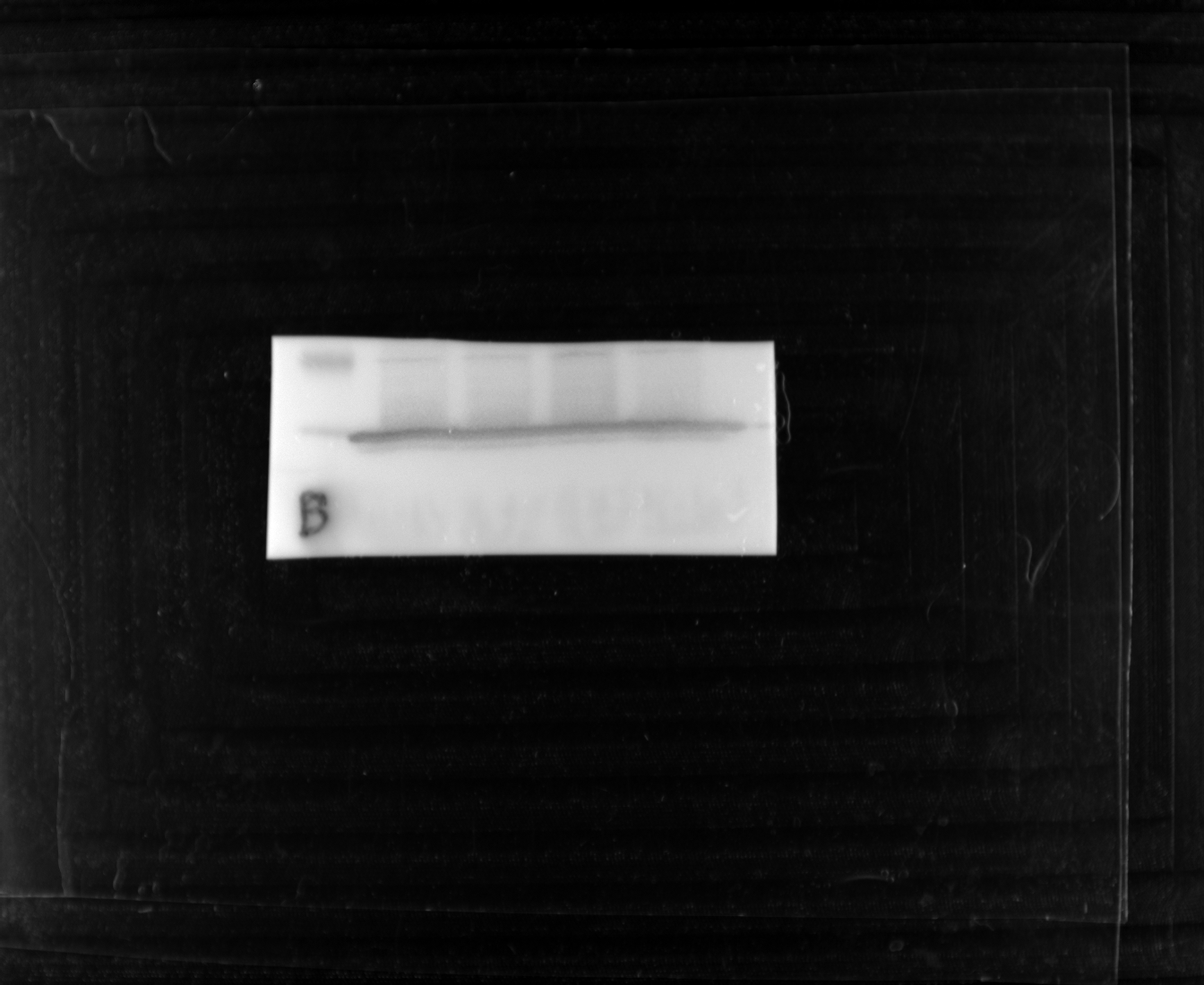

Supplement: Supplementary file 4 — Supplementary Material 4. [file 424_2026_3191_MOESM4_ESM.png]

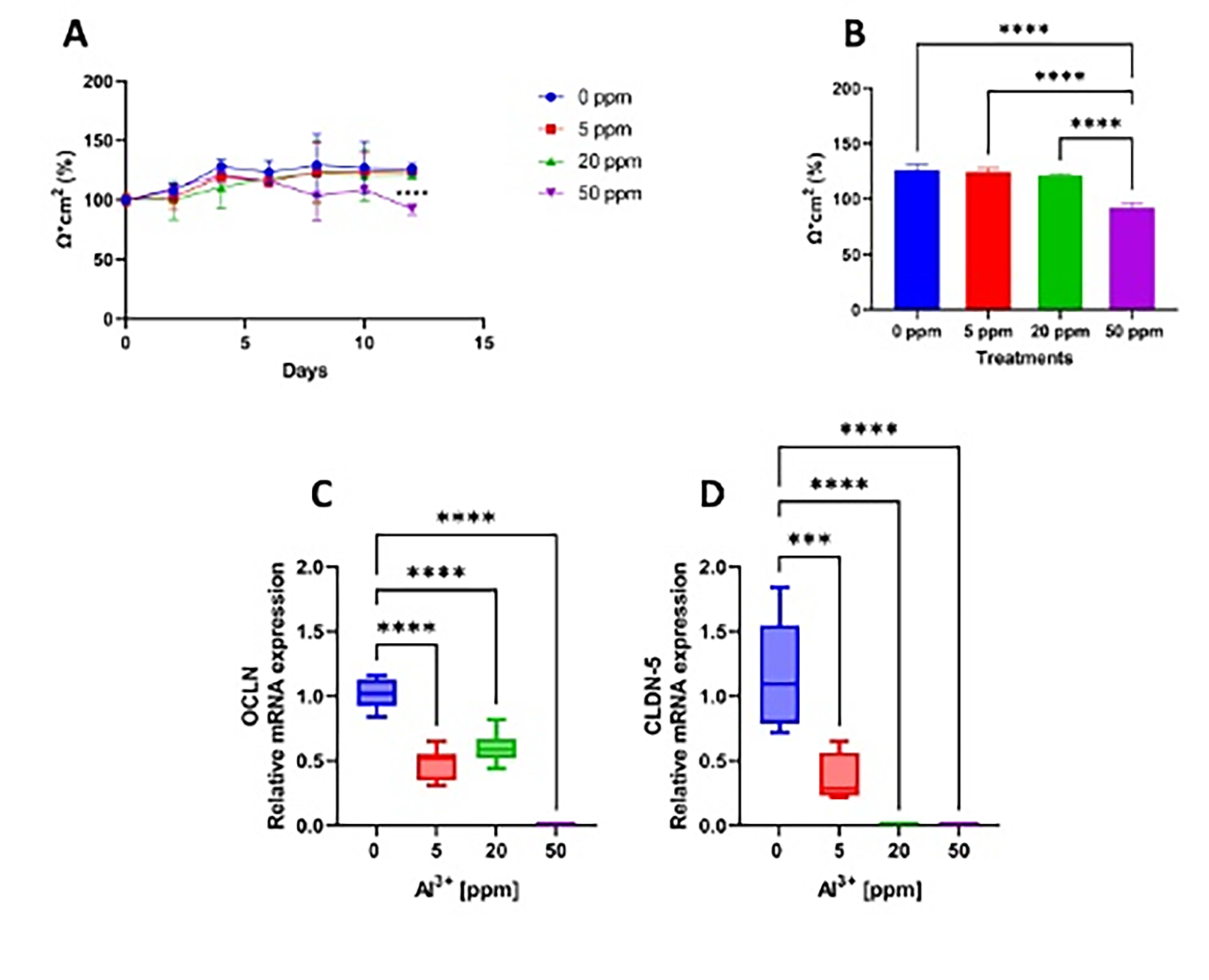

Supplement: Supplementary file 5 — Supplementary Material 5. [file 424_2026_3191_MOESM5_ESM.png]

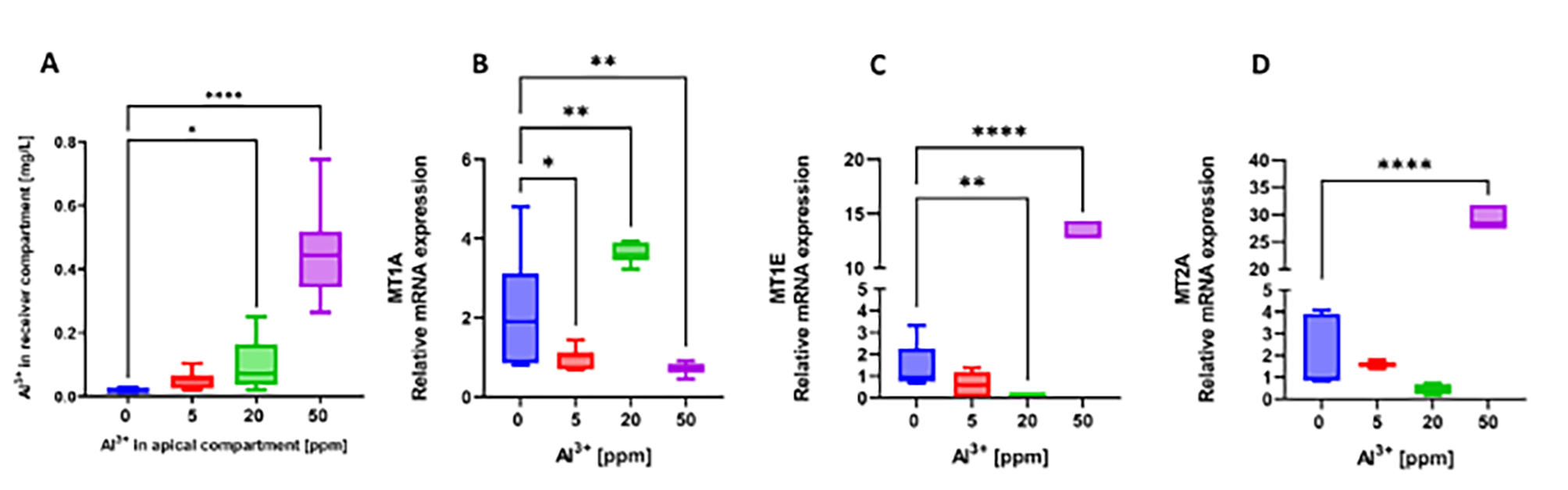

Supplement: Supplementary file 6 — Supplementary Material 6. [file 424_2026_3191_MOESM6_ESM.png]

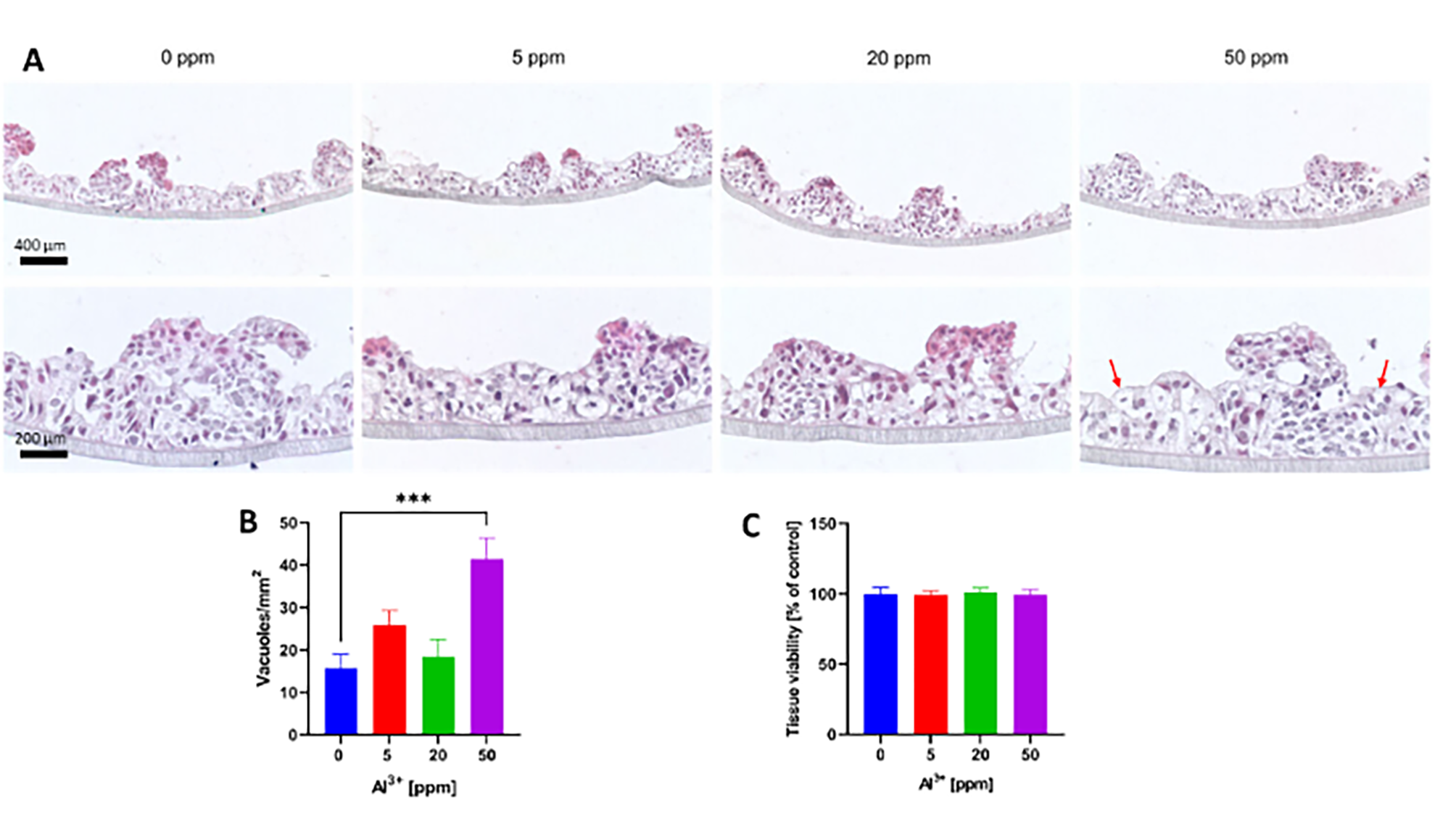

Supplement: Supplementary file 7 — Supplementary Material 7. [file 424_2026_3191_MOESM7_ESM.png]
